# Factors influencing the practice of exclusive breastfeeding among nursing mothers in a peri-urban district of Ghana

**DOI:** 10.1186/s13104-017-2774-7

**Published:** 2017-09-07

**Authors:** Kofi Akohene Mensah, Enoch Acheampong, Francis Owusu Anokye, Paul Okyere, Emmanuel Appiah-Brempong, Rose Odotei Adjei

**Affiliations:** 1 Department of Health Policy, Management and Economics, School of Public Health, Kwame Nkrumah University of Science and Technology, Kumasi, Ghana; 2 Centre for Disability and Rehabilitation Studies, Department of Community Health, Kwame Nkrumah University of Science and Technology, Kumasi, Ghana; 3 Department of Health Education and Promotion, School of Public Health, Kwame Nkrumah University of Science and Technology, Kumasi, Ghana

**Keywords:** Exclusive-breastfeeding, Ghana, Nursing mothers, Infant, Health

## Abstract

**Background:**

Exclusive breastfeeding (EBF) is one of the optimal infant and young child feeding practices. Globally, <40% of infants under 6 months of age are exclusively breastfed. In Ghana, 63% of children <6 months are exclusively breastfed which is far less than the 100% recommended by the United Nation Children Emergency Fund. This study was carried out to find out the factors that influence the practice of exclusive breastfeeding in the district.

**Methods:**

A cross-sectional quantitative study was conducted using structured questionnaires. A convenience sampling technique was employed to select 380 nursing mothers who attended postnatal care at the postnatal clinic in all the 13 health facilities with child welfare clinics (both public and private) and were available on the day of data collection. Data were analysed using frequency and CHISQ tables.

**Results:**

There was a significant association between socio-demographic characteristics of mothers such as age (p = 0.129), religion (p = 0.035) type of employment (p = 0.005) and the practice of exclusive breastfeeding. Again, there was significant relationship between mothers’ knowledge on EBF in terms of sources of information about EBF (p = 0.000), steps taken by mothers who perceived not to have breast milk (p = 0.000), some medical conditions of nursing mothers (p = 0.000) and the practice of EBF.

**Conclusion:**

Most nursing mothers use infant formula feeds as either supplement or substitute for breast milk based on their perception that breast milk may not be sufficient for the babies despite the high cost of these artificial milk. This puts the babies at a higher risk of compromised health and malnutrition which has the potential of increasing infant mortality. Most mothers are not practicing exclusive breastfeeding because their spouses and family members do not allow them.

**Electronic supplementary material:**

The online version of this article (doi:10.1186/s13104-017-2774-7) contains supplementary material, which is available to authorized users.

## Introduction and background

Exclusive breastfeeding (EBF) is one of the optimal infant and young child feeding practices. EBF is feeding infants (0–6 months of age) exclusively with breast milk for the first 6 months [1]. Such children may take only Oral Rehydration Salt (ORS), drops, and syrups (vitamins, minerals, and medicines) in addition to their mothers’ milk [1, 2]. Goal four of the eight Millennium Development Goals is entirely devoted to reducing child mortality by two-thirds between 1990 and 2015 [2]. Progress in many African countries is insufficient in achieving this goal. As a result, the World Health Assembly passed a resolution recommending exclusive breastfeeding for the first 6 months of life as part of the initiative to reduce infant mortality [1, 2].

Globally, <40% of infants under 6 months of age are exclusively breastfed. This is expected to increase to 50% by 2025. However, little is being done to give greater priority to increasing the rates of exclusive breastfeeding, despite repeated and emphatic agreement of its benefits. Although the rates of EBF for the past two decades have been increasing, it is still a long road to reaching the world’s 100% coverage target recommended by UNICEF. This is evident in the current low prevalence of EBF in much of the developing world especially in West and Central Africa which happens to have one of the highest rates of infant malnutrition in the world [3]. The global EBF rate for infants aged below 6 months between the years 2000 and 2007 was 38%. Within the same time, only 23% of infants <6 months were breastfed exclusively in West and Central Africa while a slightly higher rate (26%) was recorded in the Middle East and North Africa. Exclusive breastfeeding rates of 39, 43 and 44% were observed in Eastern and Southern Africa; East Asia and the Pacific; and South Asia respectively [4].

Ghana’s EBF rate has risen steadily from 7% in 1993 to 31% in 1998 and then to 53% in 2003 The findings of the 2008 Ghana Demographic and Health Survey (GDHS) suggest that the percentage of Ghanaian children ever breast-fed is between 97–98%. Within the same period, the percentage of infants who initiated breastfeeding within 1 h after delivery was 52%, a 6% increase over the 2003 figure of 46%. Additionally, 82% of infants aged <2 months were reported to have been breast-fed exclusively [5]. Conversely, only 49% of infants were still being breast-fed exclusively by 4–5 months. Furthermore, a total of 63% of infants below 6 months were breast-fed exclusively in 2008. Though this was an improvement upon the 2003 rate of 53%, it was still below the 100% coverage target. The results of the recent Multiple Indicator Cluster Survey (MICS) (2011) suggest that the rate of EBF has declined from 63 to 46% [5]. In other part of Ghana such as Volta Region, suboptimal feeding which causes death in children <6 months is noted to be too high [5, 6].

In view of the continuous low level of exclusive breastfeeding among mothers in Ghana, this study was carried out to ascertain the factors that influence the practice of exclusive breastfeeding.

## Methods

The study employed descriptive and analytical cross-sectional study. The study population was nursing mothers in the post-partum period in the Sekyere—South District of Ghana. The inclusion and exclusion criteria for selecting participants were; biological mothers with single babies who reside in Sekere-South District and showed interest in participating were recruited. Only mothers with infants between the ages of 1–6 months were included. In terms of exclusion criteria, biological mothers who did not reside in the district, mothers with multiple babies (twins), mothers with babies more than 6 months, mothers who were not breastfeeding at all due to personal choice or medical condition that interferes with breastfeeding such as mastitis were excluded from the study.

From a population of 3760 (nursing mothers within the district who had attended any of the facilities used for the study) at an expected frequency or response distribution of 50%, a confidence limit or margin of error of 5% at a confidence level of 95%, the sample size of the study was calculated as 349 using Epi info version 7.0.8.3. Using non-response rate of 10%, the total sample size used for the study was 380.

All mothers who attended postnatal clinic in all the 13 health facilities with child welfare clinics (both public and private) and available on the day of data collection were conveniently considered. In this case, the researchers visited each facility on postnatal clinic days. All mothers who were available during the first visit at each facility were interviewed with their consent. Subsequent visits were made to each facility till the total sample size was attained. Structured questionnaire containing close and open questions was used to gather information from the study participants (Additional file 1). The essence of the study was explained to participants using an information sheet. The questionnaire was pre-tested on a sample of 10 respondents at Effiduasi District hospital in the Sekyere West District. Data were analysed using frequency and CHISQ tables. Statistical significance for all testing was set as 0.05. In the analysis of the various factors that influence exclusive breast feeding among mothers within the district, bivariate analyses were used to assess any relationship between mothers practicing EBF and the various factors influencing the practice.

## Results

### Socio-demographic characteristics of respondents

More than half of the respondents (60.8%) were within the age range of 26–35 years, 36.3% were between 15–25 years while 2.9% were 35 years and above. Majority of the mothers (84.7%) were Akans. In terms of religion, Christians formed majority (85.3%), majority of the mothers were working in private institutions (80.8%) and <50% (49.5%) of the mothers were self-employed. Most of the participants had exactly or <5 children (93.7%) while the remaining (22.3%) had more than 5 children.

### Factors influencing exclusive breastfeeding

The factors influencing exclusive breastfeeding were assessed in terms of socio-demographic factors, and mothers’ knowledge on exclusive breastfeeding.

### Socio-demographic characteristics of the mothers and exclusive breastfeeding

Less than 16% of the mothers (15.53%) whose age fell between 26–35 years practiced exclusive breastfeeding, 8.42% of those below 26 years and few of them (1.32%) above 35 years practiced exclusive breastfeeding respectively. However the difference was not statistically significant (p = 0.129). Also, a significant proportion of mothers (14.47%) whose primary language was Twi practiced exclusive breastfeeding as compared with those whose spoke other languages (p = 0.000). The association between mothers level of education, number of births, employment status, spouse occupation, and the practice of exclusive breastfeeding was statistically significant as detailed in Table 1. Additionally, a proportion of mothers (14.47%) who were Christians practiced exclusive breastfeeding as compared with those who belonged to other denominations (Table 2). The difference was statistically significant (p = 0.035). There was an association between housing type, number of people in a household and the practice of exclusive breastfeeding.

**Table 1:** Socio-demographic characteristics of participants. *Source* Author’s field work (2015)

Variables	Frequency (N = 380)	Percent (%)
Age (years)		
15–25	138	36.3
26–35	231	60.8
Above 35	11	2.9
Primary language		
Twi	318	83.7
Hausa	23	6.0
English	4	1.1
Kotokoli	35	9.2
Highest level of education		
No formal education	1	0.3
Primary	24	6.3
J.H.S/middle school	160	42.1
S.H.S/vocational/technical	151	39.7
Tertiary	44	11.6
Marital status		
Single	107	28.2
Married	269	70.8
Divorced	4	1.1
Tribe		
Akan	322	84.7
Hausa	23	6.1
Kotokoli	35	9.2
Number of births		
1–5	356	93.7
5–10	22	5.8
Employment status		
Full time employed	105	27.6
Part-time	2	0.5
Self-employed	188	49.5
Unemployed	85	22.4
Type of employment (N = 309)		
Private	250	80.8
Region		
Christian	324	85.3
Moslem	52	13.7
Traditional	4	1.1

**Table 2:** Relationship between socio-demographic factors and exclusive breastfeeding. *Source* Author’s field work (2015)

Characteristic	Duration of breast feeding (in months) N = 380				χ^2^ (p value)
	<2 n (%)	2–4 n (%)	5–7 n (%)	8–12 n (%)	
Age (years)					
15–25	85 (22.37)	32 (8.42)	21 (5.53)	0 (0.00)	9.8897 (0.129)
26–35	124 (32.63)	59 (15.53)	44 (11.58)	4 (1.05)	
Above 35	4 (1.05)	2 (0.53)	5 (1.32)	0 (0.0)	
Highest level of education					
No formal education	0 (0.00)	0 (0.00)	1 (0.26)	0 (0.00)	59.0999 (0.000)
Primary	5 (1.32)	2 (0.53)	17 (4.47)	0 (0.00)	
J.H.S/middle school	92 (24.21)	41 (10.79)	23 (6.05)	4 (1.05)	
Secondary/vocational/technical	86 (22.63)	42 (11.05)	23 (6.05)	0 (0.00)	
Tertiary	30 (7.89)	8 (2.11)	6 (1.58)	0 (0.00)	
Religion					
Christian	190 (50.00)	75 (19.74)	55 (14.47)	4 (1.05)	13.5592 (0.035)
Moslem	19 (5.00)	18 (4.74)	15 (3.95)	0 (0.00)	
Traditional	4 (1.05)	0 (0.00)	0 (0.00)	0 (0.00)	
Tribe					
Akan	195 (51.32)	66 (17.37)	57 (15.00)	4 (1.05)	30.6940 (0.000)
Hausa	6 (1.58)	8 (2.11)	9 (2.37)	0 (0.00)	
Kotokoli	12 (3.16)	19 (5.00)	4 (1.05)	0 (0.00)	
Number of births					
1–5	203 (53.42)	93 (24.47)	56 (14.74)	4 (1.05)	34.9936 (0.000)
5–10	8 (2.11)	0 (0.00)	14 (3.68)	0 (0.00)	
Above 10	2 (0.53)	0 (0.00)	0 (0.00)	0 (0.00)	
Type of employment					
Private	136 (35.79)	55 (14.47)	57 (15.00)	2 (0.53)	18.5756 (0.005)
Public	29 (7.63)	22 (5.79)	6 (1.58)	2 (0.53)	
Not applicable	48 (12.63)	16 (4.21)	7 (1.84)	0 (0.00)	

### Mothers’ knowledge on EBF and the practice of exclusive breastfeeding

Mothers knowledge on EBF and its practiced is presented in Table 3 which shows that the source of information on exclusive breastfeeding was a major factor influencing its practice. A significant proportion of the mothers (97.1%) had information on exclusive breastfeeding from health facilities followed by media (1.8%) and the minority (1.1%) had it from TBAs. Also, most of the mothers (74.2%) deemed it appropriate to initiate exclusive breastfeeding within 30 min after birth as opposed by minority of them (0.5%) who thought exclusive breastfeeding should be initiated after 48 h. Also, artificial feeding for babies whose mothers’ breast milk production was perceived to be inadequate was supported by the majority of the respondents (83.4%) whilst a few (1.8%) stated that they would report to their doctors or nurses for advice. The difference was statistically significant (p = 0.000). Moreover, 41.6% of the mothers said that breast milk alone is not sufficient for babies within 5–6 months whilst 4.2% said breast milk insufficiency comes after 6 months and above.

**Table 3:** Mothers’ knowledge on EBF and the practice of exclusive breastfeeding. *Source* Author’s field work (2015)

Characteristic	Duration of breast feeding (in months) N = 380				χ^2^ (p value)
	<2 n (%)	2– 4 n (%)	5–7 n (%)	8–12 n (%)	
Source of information about exclusive breastfeeding					
Health facility	209 (55.00)	93 (24.47)	63 (16.58)	4 (1.05)	34.6065 (0.000)
TBA	4 (1.05)	0 (0.00)	0 (0.00)	0 (0.00)	
Media	0 (0.00)	0 (0.00)	7 (1.84)	0 (0.00)	
Appropriate time to initiate breastfeeding					
Within 30 min	159 (41.84)	65 (17.11)	54 (14.21)	4 (1.05)	9.5269 (0.390)
1–12 h	40 (10.53)	22 (5.79)	12 (3.16)	0 (0.00)	
24–48 h	14 (3.68)	4 (1.05)	4 (1.05)	0 (0.00)	
After 48 h	0 (0.00)	2 (0.53)	0 (0.00)	0 (0.00)	
Steps taken by mothers who perceived not to have breastmilk					
Continue breastfeeding	17 (4.47)	0 (0.00)	29 (7.63)	0 (0.00)	
Stop breastfeeding	8 (2.11)	0 (0.00)	2 (0.53)	0 (0.00)	86.3647 (0.000)
Add artificial food	188 (49.47)	89 (23.42)	36 (9.47)	4 (1.05)	
Will report to Dr./nurse	0 (0.00)	4 (1.05)	3 (0.79)	0 (0.00)	
Medical conditions that can prevent breastfeeding					
Cracked nipple	5 (1.32)	6 (1.58)	8 (2.11)	0 (0.00)	123.4578 (0.000)
Mastitis	135 (35.53)	41 (10.79)	12 (3.16)	0 (0.00)	
Engorged breast	12 (3.16)	6 (1.58)	11 (2.89)	0 (0.00)	
Breast cancer	52 (13.68)	20 (5.26)	6 (1.58)	0 (0.00)	
Not applicable	9 (2.37	20 (5.26)	33 (8.68)	4 (1.05)	
What should be given to babies immediately after a safe delivery					
Water	16 (4.21)	12 (3.16)	2 (0.53)	0 (0.00)	6.0371 (0.110)
Breast milk	197 (51.84	81 (21.32)	68 (7.89)	4 (1.05)	
Importance of the first yellowish breast milk					
Protects the child from diseases	157 (41.32)	76 (20.00)	61 (16.05)	4 (1.05)	8.9070 (0.179)
Contains adequate food	12 (3.16)	6 (1.58)	2 (0.53	0 (0.00)	

Significantly, most of the mothers (82.6%) were of the view that some medical conditions could prevent the practice of exclusive breastfeeding whilst 17.4% thought otherwise. Some of the medical conditions that could prevent EBF according to the respondents included mastitis (59.8%), breast cancer (24.8%), engorged breast (9.2%) and cracked nipple (6.0%). The difference was statistically significant (p = 0.000). Almost all the respondents (92.1%) agreed that breast milk was what should be given to babies after safe delivery in which 82.7% believed that the first yellowish milk was important in protecting the child against diseases and 17.2% also said it contains adequate food nutrients for healthy growth and development. Only few (17.3%) said the first yellowish milk was not important because it is dirty and must be thrown away. However, the difference was not statistically significant.

## Discussions

Mothers who are employed in the public and private sectors are less likely to practice EBF as compared to self-employed mothers. Self-employed mothers have their own schedule of work and have enough time for their babies, hence the practice of exclusive breastfeeding. These findings are in line with a study conducted by Field et al. [7] which showed that majority of the mothers who were self-employed practiced exclusive breastfeeding [7].

Majority of the respondents (80.5%) earn income less than GHC 500 yet they could afford the infant feeds. This contradicts what has been reported in sub-Saharan Africa where most African mothers cannot afford infant formula milk, and even if it is provided free there are often no mechanisms to sustain the supply [8]. This presupposes that the use of infant formula for infants <6 months goes beyond affordability. The chance of them buying these infant formula milk may not necessarily be determined by the cost but the perceived benefit of using them as supplements or substitute for breast milk especially when breast milk is perceived to be insufficient. Majority of the respondents (75.0%) supported the idea that breast milk insufficiency attracts them in buying infant feeds. Early introduction of complementary feeding for infant <6 months might pose financial challenges to the mothers coupled with managing complications that might arise in caring for these infants. These complications may include the vicious cycle of diarrhea and malnutrition which can result in increased infant mortality. It has been reported in other places that early introduction of other foods or liquids could result in diarrhoea, otitis media and other infections which may compromise the nutritional and general health status of the infants [1, 2].

Also, most of the mothers (81.3%) reported that their jobs allowed them to practice EBF whilst 18.7% stated that their jobs did not allow them to practice EBF. This is probably because majority of the respondents (80.8%) were privately employed. This might give them adequate time to breastfeed. Even within the public sector, nursing mothers have a period of 90 days maternity leave that could give them the opportunity to practice EBF at least for the first 3 months [5, 6]. However in practice, <20.0% of the mothers were practicing exclusive breastfeeding for the first 6 months. Albeit, the respondents in this study did not state any clear cultural practices that influence the practice of exclusive breastfeeding, however, the followings drivers are directly behind the practice of exclusive breastfeeding; fathers, mother and father-in-laws, grandmothers and fathers and close friends of the nursing mother. Majority of the respondents (58.7%) indicated that their spouses and families refused to support the practice of exclusive breastfeeding. In Ghana, the spouses as well as the family most of the time determine whether mothers will exclusively breastfeed especially, if the mother is not economically sound and also had the problem of allowing the babies to be thirsty over a 6 month period [5, 6].

The influential role of spouses and family members as found in this present study supports finding in the Northern part of Malawi [9]. More often than not, information on EBF recommendations is primarily tailored to meet the needs of breastfeeding mothers as if they live in isolation from other family members. Such approach consequently creates disparities in levels of understanding between breastfeeding mothers and their relatives; and this is especially true for paternal grandmothers who notwithstanding their level of influence, are often left out in many public health interventions. The paternal grandmother, father, and the grandfather are basically the decision makers [9]. Considering the fact that most spouses were not supportive of the practice of exclusive breastfeeding, it would have been interesting to note the kind of information the husbands of these mothers would have given. However, the study did not include husbands whose wives took part in the study. The study could have also obtained information from the husbands as to whether they support the decisions of their wives to practice exclusive breastfeeding or not since husbands also play significant role in determining the decisions of the families. This is considered as a limitation of the study.

Majority of the mothers (97.1%) have heard of exclusive breastfeeding from health facilities. Even though knowledge on breastfeeding was high, the practice of EBF was low as confirmed by the prevalence rate of mothers practicing exclusive breastfeeding for the first 6 months. Therefore, it is clear that just having a good knowledge of EBF does not suggest that mothers would breastfeed exclusively for the first 6 months though knowledge is vital in improving EBF. This situation is evident in other African countries like Tanzania and Nigeria where knowledge on exclusive breastfeeding was high but the practice of it was low. For instance, a study conducted in Nigeria revealed that as high as 91.2% of the study participants had very good knowledge on EBF but only 37.3% of them were practicing EBF [10]. This high knowledge of EBF but low practice of EBF might be as a result of challenges mothers encounter whilst practicing exclusive breastfeeding couple with inadequate support and inappropriate information they received as a solution to overcome those challenges [10]. Therefore, intervention which would help to convert high knowledge of EBF into high practices cannot be underestimated. The use of media specifically TV advertisement as a communication tool has been proven successful in changing attitude towards infant feeding practices [5, 6]. The only current National Child Nutrition Campaign on TV in Ghana is the United States Agency for International Development (USAID) sponsored advert “Aduane Pa Ma Asetena Pa’’ (Good Food for Good Life). The advert which was launched in 2013 by the Ministry of Health and the Ghana Health Service on Ghanaian TV channels mainly focuses on appropriate and timely complementary feeding after 6 months. It does not provide much information on EBF for the first 6 months. Modification of the “Aduane Pa Ma Asetena Pa’’ advert to include more information on the benefits of EBF for the first 6 months may increase EBF rates. Also, educating caregivers and other family members such as fathers and grandmothers through TV to understand that well-nourished mothers can produce adequate amounts of breast milk to feed their infants until 6 months could contribute to higher rates in future. This is because most people are easily influence by visuals rather than words [5, 6].

## Conclusion

Most nursing mothers use infant formula feeds as either supplement or substitute for breast milk based on their perception that breast milk may not be sufficient for the babies despite the high cost of these artificial milk. This puts the babies at a higher risk of compromised health and malnutrition which has the potential of increasing infant mortality. Most mothers are not practicing exclusive breastfeeding because their spouses and family members do not allow them.

## Recommendations

1. It is recommended that authorities of the various health facilities within the district should collaborate with the National Commission for Civic Education to embark on outreach programmes targeting pregnant women, nursing mothers and their spouses within their catchment areas to provide adequate information on the need to practice exclusive breastfeeding. The outreach programmes should also focus on bringing to light the negative health implications of using artificial infant milk as substitute or supplement for breast milk.
2. The Ministry of Health and the Ghana Health Service should collaborate with the Ghana media commission and the Ghana Journalist Association to promote exclusive breastfeeding using the media through adverts and promotions.
3. It is also recommended that further studies should be conducted on the role and influence of family members and husbands of nursing mothers on the practice of exclusive breastfeeding.

## Additional file

**Additional file 1.** Questionnaire.

## Abbreviations

EBF: exclusive breastfeeding
UNICEF: United Nation Children Emergency Fund
ORS: Oral Rehydration Salt
GDHS: Ghana Demographic and Health Survey
MICS: Multiple Indicator Cluster Survey
