# Supplementary material for: Factors influencing the practice of exclusive breastfeeding among nursing mothers in a peri-urban district of Ghana
Source: BMC Res Notes. 2017 Sep 7;10:466. doi: 10.1186/s13104-017-2774-7 (PMC5590172; doi:10.1186/s13104-017-2774-7)
Supplement: Supplementary file 1 — Additional file 1. Questionnaire. [file 13104_2017_2774_MOESM1_ESM.docx]

### QUESTIONAIRE

**SECTION A SOCIO-DEMOGRAPHIC CHARACTERISTICS**

1. What is your age?

Under 15[ ] 15-25 [ ] 25-35[ ] Above 35 [ ]

1. What is your primary language? … … … … … …
2. What is the highest level of education you have completed?

No formal education [ ] Primary [ ] J.H.S/Middle school [ ]

Secondary/vocational/Technical [ ] Tertiary [ ]

1. Marital status?

Single [ ] Married [ ] Divorced [ ] Separated [ ] Widowed [ ]

1. How far do you stay from a health center?

Less than 2km [ ] 2km to 4 km [ ] More than 4km [ ]

1. Tribe ………………….
2. Number of births (Dead and Alive).....................................
3. Employment status?

Full time employed [ ] Part time employment [ ] Self-employed [ ] unemployed [ ]

1. What type of employment

Private [ ] Public [ ]

1. Spouse occupation……………………………….
2. What is your current household income status (GH¢)

Less than 500 (low) [ ] 500-1000 (Medium) [ ] Above 1000 (High) [ ]

1. Religion

Christian [ ] Moslem [ ] Traditional [ ] Others [ ]

1. Housing type

Apartment [ ] Detached [ ] Semidetached [ ]

1. Number in household …………….

**SECTION B PREVALENCE OF EXCLUSIVE BREASTFEEDING**

1. When did you initiate breastfeeding after birth

Within 1 hour [ ] More than 1 hour [ ]

1. Did you give all the colostrums

Yes [ ] No [ ]

1. How long have you been breastfeeding?

Less than 2 months [ ] 2-4months [ ] 4-6months [ ] More than 6 months [ ]

1. Are you still practicing or did you practice exclusive breastfeeding?

Yes [ ] No [ ]

1. Did you give prelacteal feeds in addition to the breast milk?

Yes [ ] No [ ]

1. What was the duration of your previous breastfeeding?

……………….

1. How long did you practice exclusive breastfeeding for your previous birth?

Less than 2 months [ ] 2-4months [ ] 4-6months [ ]

**SECTION C: FACTORS INFLUENCING EXCLUSIVE BREASTFEEDING**

Knowledge on exclusive breastfeeding

1. Where did you get information about the exclusive breastfeeding?

At antenatal clinic [ ] At delivery at home [ ] Before discharge [ ] on TV [ ]

1. When do you think it is appropriate to initiate breastfeeding after a safe delivery?

Yes [ ] No [ ]

1. What would you do if your baby was under four months of age and you thought he was not getting enough breast milk?

Continue breastfeeding [ ] Stop Breastfeeding [ ] Add artificial food [ ] Other [ ]

1. How long do you think breast milk alone, without even water, is sufficient for a baby?

……………………………

1. A. do you think there are some medical conditions that can prevent you from practicing exclusive breastfeeding?

Yes [ ] No [ ]

B. if yes in A, what do you think are some of the examples

Crack Nipple [ ] Mastitis [ ] Engorged breast [ ]

…………………………………………………………………..

1. What should be given to a baby immediately after a safe delivery?

Water [ ] breast milk [ ] Other [ ]

1. A. Is there any importance in feeding a baby the first yellowish milk?

Yes [ ] No [ ]

B. If yes, what are some of the importance?

………………………………………………………………………………………………

**Socioeconomic factors influencing exclusive breastfeeding**

a. Can you afford to practice prelacteal or suboptimal feeding of your baby?

Yes [ ] No [ ]

b. Does that attract you to practice suboptimal feeding?

Yes [ ] No [ ]

1. Does your job allow you to breastfeed adequately?

Yes [ ] No [ ]

1. a. Will continuous breastfeeding affect your job?

Yes [ ] No [ ]

b. If yes above, how? ………………………………………………………………………………………………………………………………………………………………………………………………

1. How does your job affect the practice of exclusive breastfeeding?

Reduces

1. **Sociocultural factors**
2. a. Does your culture affect the practice of exclusive breastfeeding in any way?

Yes [ ] No [ ]

b. If yes, how?

………………………………………………………………………………………………………………………………………………………………………………………………

1. a. Do you get support from your spouse in the practice of exclusive breastfeeding?

Yes [ ] No [ ]

b. If yes, how?

………………………………………………………………………………………………………………………………………………………………………………………………

1. a. Do you get support from your other family members?

Yes [ ] No [ ]

b. If yes, how?

………………………………………………………………………………………………………………………………………………………………………………………………

1. What will be the consequence if you breastfeed exclusively?

………………………………………………………………………………………………………………………………………………………………………………………………
